# Development and Validation of the Latvian Version of the Orofacial Esthetic Scale in Dental Patients with Aesthetic, Functional and No Treatment Needs

**DOI:** 10.3390/medicina61122180

**Published:** 2025-12-08

**Authors:** Mara Gaile, Simona Skrivele, Pernilla Larsson, Oskars Radzins, Una Soboleva, Christel Larsson

**Affiliations:** 1 Department of Prosthetic Dentistry, Riga Stradins University, LV-1007 Riga, Latvia; una.soboleva@rsu.lv; 2 Department of Conservative Dentistry and Oral Health, Riga Stradins University, LV-1007 Riga, Latvia; simona.skrivele@rsu.lv; 3 Department of Prosthetic Dentistry, Malmӧ University, SE-205 06 Malmö, Sweden; pernilla@framtand.se (P.L.); christel.larsson@mau.se (C.L.); 4 Department of Orthodontics, Riga Stradins University, LV-1007 Riga, Latvia; oskars.radzins@rsu.lv

**Keywords:** aesthetic dentistry, orofacial aesthetics, psychometric properties, questionnaire

## Abstract

*Background and Objectives*: This study was conducted in order to develop and validate the Latvian version of the Orofacial Aesthetic Scale (OES-LV) and to assess its psychometric properties in patients with aesthetic, functional or no treatment needs. *Materials and Methods*: The English version of the OES was translated into Latvian following international guidelines for establishing cultural equivalency of instruments. The test group consisted of 101 subjects comprised of those without treatment requirement, with functional impairment (tooth loss) and with aesthetic treatment needs. Internal consistency, test–retest reliability and convergent validity were investigated. Responsiveness was not tested in the current study. *Results*: The test–retest assessment of this study was performed on 31 subjects and showed intra-class correlation coefficients ranging from 0.80 to 0.81, which was considered to be good. Cronbach’s α was 0.91, demonstrating the strong internal consistency of the scale. Spearman’s rank correlation coefficients between the OHIP and OES scores varied across subgroups, ranging from −0.35 to −0.57 and showed a negative correlation between OES-LV and selected OHIP items. *Conclusions*: The Latvian OES demonstrated strong psychometric properties, supporting its use in assessing self-perceived orofacial aesthetics, clinical research, prosthodontic evaluation and dental education. Further studies on responsiveness are recommended.

## 1. Introduction

With general improvements in oral health, a good aesthetic outcome is becoming more important for a treatment to be considered successful [1]. Dental aesthetics have an impact on individuals’ self-esteem and quality of life, especially for young adults [2]. General dentists, dental specialists and laypeople perceive specific dental aesthetic deviations differently [3] and have varying expectations regarding treatment outcome [4]. Therefore, the focus has shifted from specialist-based evaluations to patient-reported outcome measures (PROMs). PROMs are increasingly being used as a measure of treatment success in both medicine and dentistry [5].

Dental aesthetics is an important component of oral health-related quality of life (OHRQoL). Although the Oral Health Impact Profile (OHIP) [6], a widely used oral OHRQoL questionnaire, has been adapted and validated for use in Latvian, it does not adequately capture the specific aesthetic dimensions of the orofacial region that are particularly relevant in prosthodontic treatment. When applied to patients with concerns about orofacial appearance, the OHIP typically reflects a variable but generally moderate impact on OHRQoL [7], underscoring the need for more targeted aesthetic assessment tools.

The Orofacial Esthetic Scale (OES) was developed precisely to address these aspects. It is a brief patient-reported outcome measure designed specifically for prosthodontic patients, assessing orofacial aesthetic impacts and demonstrating good reliability and validity [8]. The OES was originally developed in Swedish and English [9], and subsequent versions, including Dutch, Finnish and Croatian, have been created in response to international research interest [10,11,12]. However, no Latvian version has been available to date. The development of a Latvian adaptation therefore represents a valuable addition to the expanding set of international OES versions, supporting its application in prosthodontic and aesthetic dentistry research and addressing an important gap in cross-cultural adaptation studies.

## 2. Materials and Methods

### 2.1. OES Translation

OES is an eight-item instrument with seven items addressing aesthetic impacts of the orofacial region and an eighth item for a global assessment. It applies an 11-point scale, with summary scores ranging from 0 (worst) to 70 (best). Answers to the first seven questions are scored on a scale from 0 (very dissatisfied) to 10 (very satisfied) and added as a summary evaluation score. Item number eight (global assessment) is evaluated separately from 0 to 10 and is an overall impression score.

Two forward translations of the English version of OES were performed independently by two prosthodontic specialists whose native language was Latvian. Translations were compared, synthesized together and back-translated (the forward–backward method) by an independent translator whose native language was English. When the translated version was compared to the original Orofacial Esthetic Scale English version (OES-E), no discrepancies were present between the two versions.

**Figure 1:**
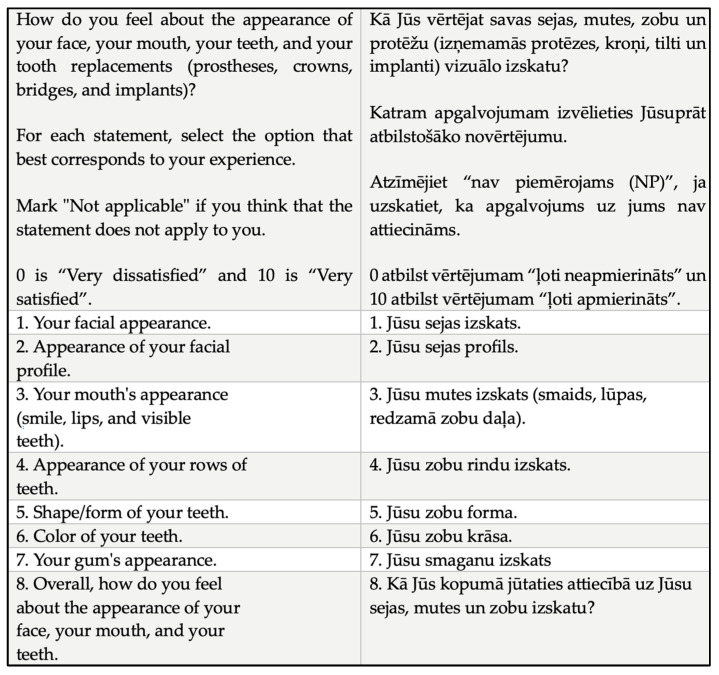
The English and Latvian versions of the Orofacial Esthetic Scale.

Evaluation of the final translation was performed by the research team and laypeople following minor revisions and changes. The final version (OES-LV) was developed (see Figure 1).

### 2.2. Patient Sample

This study was approved by the Riga Stradins University (RSU) Research and Ethics Committee (permit number 2-PĒK-4/554/2024). Adults aged 18 years or older who attended the RSU Institute of Stomatology between January and March 2025 and demonstrated sufficient proficiency in the Latvian language to complete the questionnaire independently were invited to participate.

The inclusion criteria were as follows: 1. patients who attended an initial consultation at the Aesthetic Dentistry Clinic or Prosthodontics Department; 2. patients who underwent a dental hygiene appointment or a follow-up visit after completion of prosthetic treatment, including both removable and fixed prosthetic restorations. Second-year preclinical dental students from RSU were also invited to participate as a control group.

Patients with communication difficulties, inadequate Latvian language skills, acute dental problems, temporomandibular disorders, or those undergoing orthodontic treatment were excluded. No additional screening procedures were applied, ensuring that the sample represented the typical patient population treated in a university-based dental clinic.

The study sample consisted of 101 participants (43 males and 58 females) with a mean age of 38.25 years (SD = 16.64; range: 20–85 years). All participants were divided into 4 groups: 1. aesthetically impaired (AI) patients who required direct aesthetic restorations (n = 24); 2. functionally impaired (FI) patients who required tooth replacement and/or crowns (n = 25); 3. patients who had previously received prosthodontic treatment (TC) and were attending control visits (n = 18); and 4. dental students with no treatment needs (SC) as a control group n = 34.

A paper-based questionnaire was distributed to patients prior to their dental appointments and to dental students before their scheduled prosthodontics classes; the questionnaire was re-administered to a subset of participants after an interval of 1–2 weeks. The OES questionnaires with missing responses were excluded from further statistical analysis. No missing responses remained in the final dataset, as any incomplete OES questionnaires were excluded at the time of data collection.

### 2.3. Reliability

To assess the reliability, internal consistency and test–retest reliability were evaluated. To evaluate test–retest reliability, testing was repeated on some of the participants (n = 31) 1 to 2 weeks after the initial questionnaire, with no dental intervention performed between the two assessments. Intra-class correlation coefficients (ICCs) were calculated for summary scores, as well as for overall impression scores. An ICC > 0.75 was considered to describe excellent reliability [13,14].

Internal consistency was evaluated by Cronbach’s alpha values [15]. Corrected item–total correlations were calculated.

### 2.4. Validity

Convergent validity of the OES was evaluated using the Oral Health Impact Profile (OHIP) as the reference instrument, following a previously established approach [8,16]. OHIP is a reliable and valid oral health-related quality of life (OHRQL) instrument that is available in Latvian [17].

Three OHIP-49 items related to orofacial aesthetics (Item 3, 22 and 31) were added to the questionnaire:

1. Have you noticed a tooth that does not look right? (Item 3)
2. Have you felt uncomfortable about the appearance of your teeth, mouth or dentures? (Item 22)
3. Have you avoided smiling because of problems with your teeth, mouth or dentures? (Item 31)

Spearman’s rank correlation coefficients were calculated to examine the association between OES summary scores and OHIP aesthetics item summary scores for the total study sample (n = 101), as well as for the four subgroups (AI, FI, SC and TC). Responsiveness was not evaluated in the present study.

### 2.5. Group Comparisons

A combination of Q-Q plot inspection and the Shapiro–Wilk test was performed for all survey scores to assess normality. For scores meeting parametric assumptions, group differences were evaluated using a one-way ANOVA; otherwise, the non-parametric Kruskal–Wallis test was applied. When the omnibus test result was significant, differences between groups were examined with a two-sample t-test or a Mann Whitney U-test, respectively, with a Bonferroni correction applied in all cases. The level of significance was set at 0.05. All statistical computations were conducted using R (version 4.3.2).

## 3. Results

### 3.1. Translation

The translation process was deemed satisfactory, and the research team reached full agreement on the translation of all items. However, a couple of patients required clarification regarding item no. 2, “Appearance of your facial profile.” They initially assumed they should lower their score if they were dissatisfied with features such as their nose or wrinkles around their forehead and eyes. To address this, an explanation was added, specifying that the focus should be on the lower third of the face.

### 3.2. Study Participants and Scores

A total of 101 participants completed the questionnaires, with a slightly higher proportion of females than males. The age range was broad, and the sample can be considered representative of typical prosthodontic patients. Gender and age characteristics of the respondents are shown in Table 1.

There were five questionnaires with missing OHIP items across the entire sample—three from the SC group and two from the TC group—and these were excluded from further analyses. As the OES section of these questionnaires was completed in full, the questionnaires were retained, and only the OHIP component was excluded from the analysis. Consequently, no imputation strategies were required.

**Figure 2:**
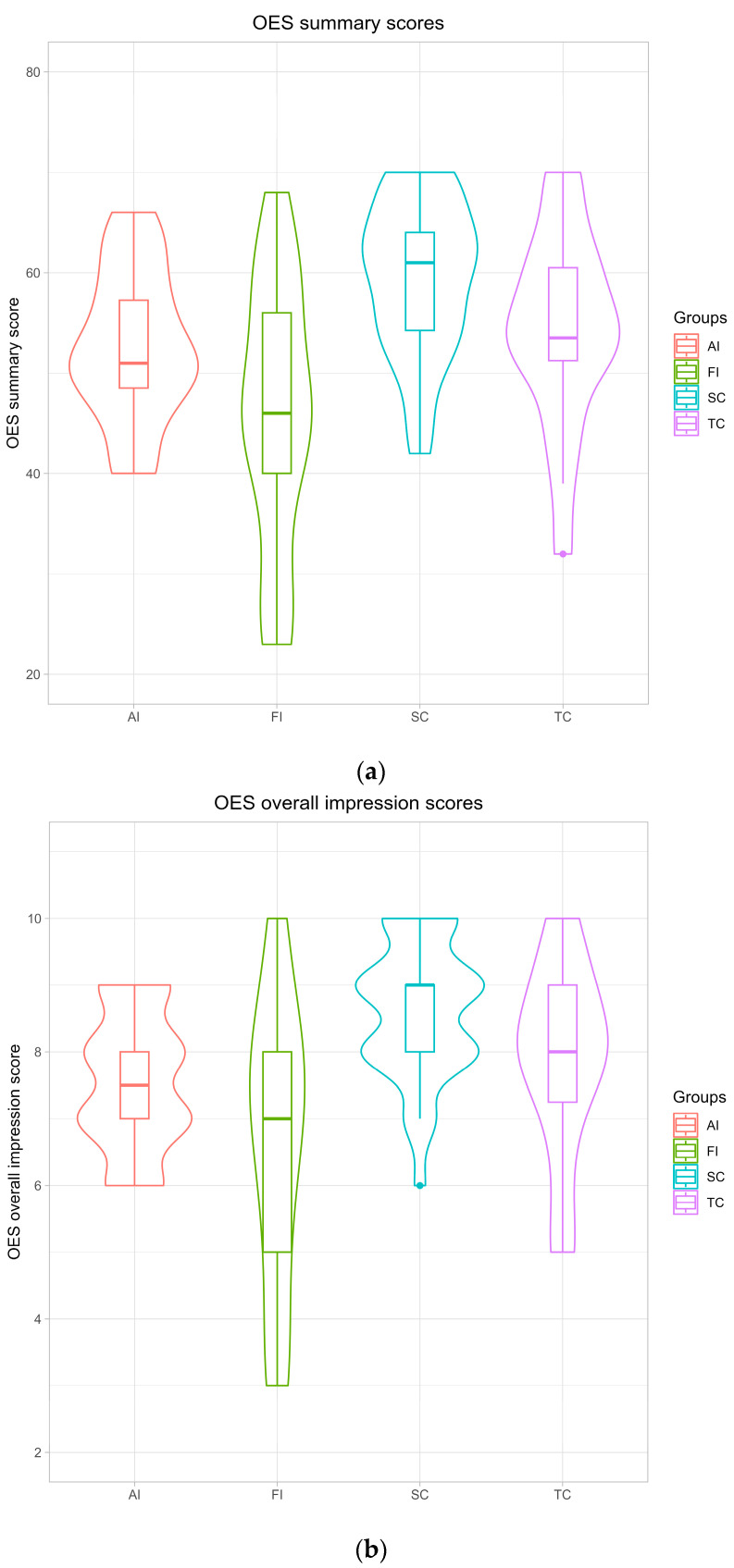
Mean OES scores: (**a**) summary scores and standard deviations; (**b**) overall impression scores and standard deviations. AI—aesthetically impaired; FI—functionally impaired; SC—dental students without treatment; and TC—previously treated patients.

Mean OES summary scores and mean overall impression scores with standard deviations for all groups are presented in Figure 2a,b. The mean OES summary and overall impression scores were comparatively higher in the SC and TC groups, followed by the AI group, whereas the FI group demonstrated comparatively lower scores on both measures.

**Figure 3:**
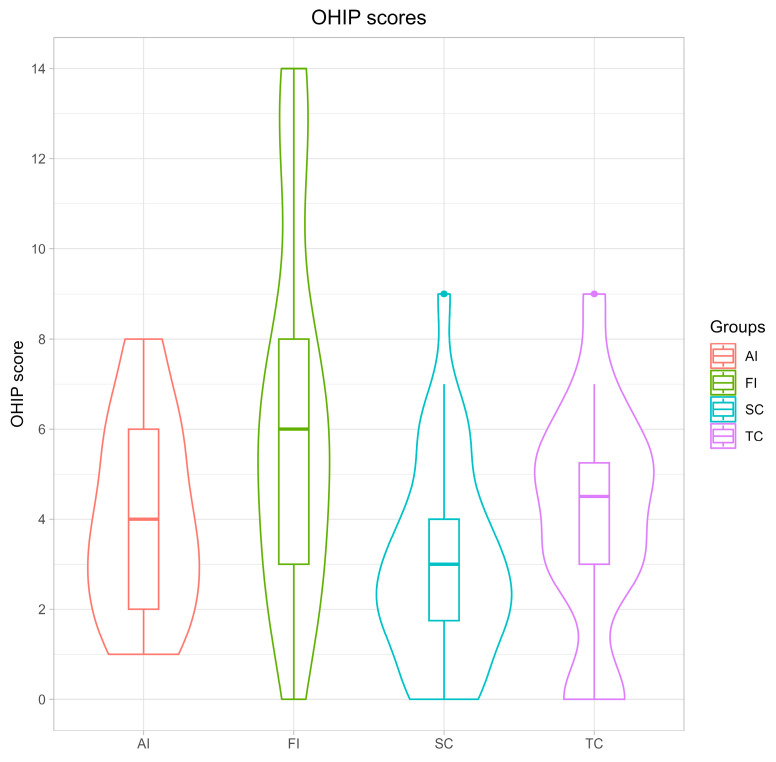
Mean OHIP scores and standard deviations. AI—aesthetically impaired; FI—functionally impaired; SC—dental students without treatment; and TC—previously treated patients.

The mean summary scores for the three OHIP items are presented in Figure 3. The student control group and the treated prosthodontic patients demonstrated comparatively lower OHIP scores, with the SC group showing the lowest values. The FI group exhibited the highest OHIP mean scores, followed by the AI group. The OES scores displayed an inverse pattern, with comparatively higher values in the groups without treatment requirements (SC and TC) and lower values in the AI and FI groups.

### 3.3. Reliability

Reliability was evaluated through assessments of internal consistency and test–retest reliability. Internal consistency was examined using Cronbach’s α coefficient, and ICCs were calculated for both the summary scores and the overall impression score. The ICCs for the OES summary scores and the overall satisfaction score are presented in Table 2, with both values exceeding 0.75. No significant differences were observed between subgroups.

Cronbach’s α was calculated to be 0.91, demonstrating the strong internal consistency of the scale, as values above 0.80 are generally considered indicative of good reliability [18]. Item-level coefficients are presented in Table 3.

### 3.4. Validity

Spearman’s rank correlation coefficient between OHIP and OES scores varied across subgroups, ranging from −0.35 to −0.57, indicating a negative association. Higher OES scores corresponded to lower OHIP scores, as expected. For the total sample, the correlation coefficient was −0.51 (95% CI: −0.65 to −0.35). Subgroup-specific values are presented in Table 4. The AI group does not show a significant correlation; however, it does not seem to be a detrimental to the entire surveyed sample, where the correlation is indeed significant. This could be explained by the limited sample size.

### 3.5. Group Comparisons

A comparison of the OES summary scores revealed large-sized statistically significant differences between the AI and SC groups (*p* = 0.007, d = 0.91) and between the FI and SC groups (*p* = 0.0003, d = 1.32) when considering all respondents. Similarly, the OES overall impression scores also showed a large effect and significant differences between the AI and SC groups (*p* = 0.003, r = 0.76) and between the FI and SC groups (*p* = 0.0005, r = 0.80). As anticipated, both the aesthetically and functionally impaired groups reported lower scores compared with the control group and prosthodontically treated patients.

Exploratory gender-specific analysis among the female respondents showed large-sized differences in OES summary scores, with significant differences between the AI and SC groups (*p* = 0.028, d = 1.00) and between the FI and SC groups (*p* = 0.036, d = 1.29). For the OES overall impression scores, significant differences and a small effect were found between the AI and SC groups (*p* = 0.013, r = 0.31), the FI and SC groups (*p* = 0.002, r = 0.23) and the FI and TC groups (*p* = 0.026, r = 0.23).

Within the male group, statistically significant large-sized differences were observed only between the FI and SC groups for the OES summary scores (*p* = 0.016, d = 1.31). No statistically significant differences were detected for the OES overall impression scores. However, these results should be interpreted cautiously due to limited power.

## 4. Discussion

This study aimed to develop a Latvian version of the Orofacial Esthetic Scale (OES-LV) and to evaluate its psychometric properties. Although the instrument demonstrated good reliability, convergent validity and internal consistency—indicating that the OES-LV is suitable for assessing self-perceived orofacial aesthetics in Latvian dental patients—several strengths and limitations of the present study should be acknowledged.

Culturally, the study group of the current study can be considered generally homogeneous, as only patients with sufficient proficiency in the Latvian language were included. It has been shown that culture and race significantly influence individuals’ aesthetic preferences regarding smile attractiveness [19]. In addition, the terminology related to dental and facial aesthetics may also differ between countries and be linguistically complex, which can further affect patients’ comprehension and communication of aesthetic concerns [20]. Cultural norms determine what is seen as attractive—whether very white, straight teeth [21], acceptance of natural variations and distinctive features such as mild misalignment [22] or mutilation and decorative modifications [23,24]. There are currently no data on how the dental aesthetic perceptions of Latvian-speaking patients compare with those of other cultures. The present study may serve as an initial contribution to this area of research.

The translation process was completed satisfactorily, and, as in a previous Croatian study [12], an additional explanation was needed for item no. 2, “Appearance of your facial profile,” to emphasize that the focus should be on the lower third of the face. Clarification was particularly required among a few older participants, who initially interpreted the question as referring to general facial appearance, including features such as forehead or eye wrinkles or the shape of the nose. Although the item wording itself was not modified, the added explanation ensured understanding among respondents.

The OES-LV employs an 11-point scale, consistent with the original version [8], whereas Croatian authors have adopted a 5-point Likert scale and recommend it for international use [12]. Other studies have likewise adapted the OES to a 5-point scale, owing to cultural conventions and parallels with educational grading systems [25]. As the Latvian education system employs an 11-point scale, it was decided to retain the originally proposed version.

In the present study, participants were categorized into four groups: three case groups consisting of individuals with different types of treatment needs or finished treatment and one control group comprising students without treatment requirements. The control group included second-year preclinical students, which may help reduce potential bias related to professional knowledge or clinical experience. The inclusion of both case and control groups was based on recommendations that such a design allows for a more robust estimation of the validity of a clinical test [26]. Similarly, case and control groups have been used in previous validation studies, including patients with and without tooth wear [10], with and without prosthetic treatment needs [27,28] or aesthetically versus functionally impaired [12,27].

Dental students are frequently used as control groups in similar studies due to their accessibility and relative homogeneity [25,29]. However, they tend to be younger than the other study groups, which represents a limitation of the present study and may potentially account for observed differences in satisfaction levels. In the current study, group comparisons showed a significantly lower OES summary and overall satisfaction scores in the functionally and aesthetically impaired groups compared with the student controls. As expected, the highest scores were observed among students, who generally lack dental treatment needs. This pattern is consistent with previous studies reporting the highest aesthetic scores in groups without treatment requirements [27,28].

A more detailed classification has been applied to prosthodontic patients in other studies—for example, specifying the type of denture used (fixed or removable, partial or complete) [12,25]—which was not addressed in the present study but may represent an important factor influencing patient satisfaction. The absence of such classification is a limitation, as fixed prostheses generally provide greater comfort, stability and aesthetic benefits compared with removable dentures, which may be associated with functional challenges or psychological discomfort. Consequently, the type of prosthetic restoration could affect self-perceived orofacial aesthetics.

The absence of responsiveness testing in the current study limits the conclusions that can be drawn about the OES-LV, as it prevents any determination of how well the instrument detects meaningful changes in orofacial aesthetics following treatment. Without an evaluation of score changes over time, the sensitivity of the OES-LV to clinical improvements or deterioration remains unknown. Responsiveness has been tested in other similar studies [12,30], and it should be evaluated for the OES-LV in future research.

The test–retest reliability of the OES-LV was good, consistent with findings from other similar studies [10,12,31].

Convergent validity was assessed using three items from the OHIP-LV and calculating their correlation with the OES-LV. As both scales operate in reverse, lower OHIP scores are expected to correlate with higher OES scores. This correlation was observed in the present study, consistent with findings from previous works [10,29].

A limitation concerning the generalizability and external validity of this study is that all participants were recruited from a single institution—the RSU Institute of Stomatology, a university-based specialist clinic—and may therefore represent more complex cases than those typically seen in the general Latvian population. The patient groups were clinically heterogeneous, reflecting the broad spectrum of conditions commonly encountered in prosthodontic practice. Similar to observations from a previous German study [31], prosthodontic patients can be considered representative of typical dental patients with multiple coexisting problems, such as caries, periodontitis, and tooth loss, and they span a wide range of age groups.

Exploratory gender-specific analyses indicated potential differences only in the female subgroup. Among male respondents, the differences in scores between the aesthetically impaired and control group were not statistically significant. This finding is consistent with other studies on patient perceptions of dental aesthetics, which suggest that females may be slightly more concerned about their dental appearance [32,33] and may also experience greater psychological impacts from aesthetic impairments compared to males [34]. However, these analyses were not prespecified, and this study was not powered for subgroup comparisons, so the findings should be interpreted cautiously and warrant further investigation in future studies.

The OES provides a structured measure of patients’ perceptions of their appearance, supporting treatment evaluation, satisfaction assessment and patient education. Score changes help assess treatment effectiveness, while item-level results clarify priorities and guide decisions. In dental education, it can help students to understand patient-cantered aesthetic assessment and integrate patient-reported outcomes into clinical decision-making. It is a practical and easy-to-use instrument for evaluating self-perceived orofacial aesthetics and may be valuable for application in the Latvian population, both in routine clinical assessments and in research and educational contexts.

## 5. Conclusions

The Latvian version of the OES demonstrated strong psychometric properties, supporting its use for assessing self-perceived orofacial aesthetics in dental patients with functional and aesthetic treatment needs, as well as clinical research, prosthodontic treatment evaluation and dental education. Further studies evaluating responsiveness are recommended.

## Figures and Tables

**Table 1:** Gender and age characteristics of the respondents with minimum, maximum, mean and standard deviation (SD) values. M—male; F—female.

				Age		
Group	Gender	N	Min	Max	Mean	SD
All	Both	101	20	85	38.25	16.64
	M	43	20	75	39.72	17.4
	F	58	20	85	37.16	16.12
Aesthetically impaired (AI)	Both	24	25	63	40.83	10.38
	M	8	31	61	43	9.9
	F	16	25	63	39.75	10.75
Functionally impaired (FI)	Both	25	22	73	49.6	13.79
	M	14	22	73	50.21	15.89
	F	11	32	68	48.82	11.26
Dental students without treatment (SC)	Both	34	20	26	21.03	1.42
	M	14	20	23	20.71	0.91
	F	20	20	26	21.25	1.68
Previously treated patients (TC)	Both	18	31	85	51.56	15.21
	M	7	37	75	53	11.65
	F	11	31	85	50.64	17.6

**Table 2:** Intra-class correlation coefficients (ICCs) characterizing the test–retest reliability assessment of the Orofacial Esthetic Scale.

	ICC (95% CI)
**OES summary**	0.80 (0.59; 0.90)
**OES overall impression**	0.81 (0.60; 0.91)

**Table 3:** Cronbach’s alpha values for the OES-LV.

Cronbach’s Alpha (If Item Deleted) (95% CI)								Corrected Item Correlation						
Overall	1	2	3	4	5	6	7	1	2	3	4	5	6	7
0.91 (0.88; 0.93)	0.89 (0.85; 0.92)	0.90 (0.87; 0.93)	0.88 (0.84; 0.91)	0.89 (0.85; 0.92)	0.89 (0.85; 0.92)	0.89 (0.85; 0.92)	0.9 (0.87; 0.93)	0.78	0.71	0.82	0.82	0.83	0.78	0.64

**Table 4:** Spearman’s rank correlation coefficients with 95% confidence intervals (CIs) for the total study sample and subgroups.

	N	Spearman’s Rank Correlation (95% CI)
**Total**	101	−0.51 (−0.65; −0.35)
**Aesthetically impaired (AI)**	24	−0.35 (−0.66; 0.06)
**Functionally impaired (FI)**	25	−0.47 (−0.73; −0.08)
**Dental students without treatment (SC)**	34	−0.57 (−0.77; −0.28)
**Previously treated patients (TC)**	18	−0.57 (−0.83; −0.10)

## Data Availability

The data that support the findings of this study are available from the corresponding author, M. Gaile, upon request.

## References

[1] Samorodnitzky-Naveh G.R.D.M.D., Geiger S.B.D.M.D., Levin L.D.M.D. (2007). Patients’ satisfaction with dental esthetics. J. Am. Dent. Assoc..

[2] Gavric A., Mirceta D., Jakobovic M., Pavlic A., Zrinski M.T., Spalj S. (2015). Craniodentofacial characteristics, dental esthetics–related quality of life, and self-esteem. Am. J. Orthod. Dentofac. Orthop..

[3] Kokich V.O., Kiyak H.A., Shapiro P.A. (1999). Comparing the perception of dentists and lay people to altered dental esthetics. J. Esthet. Restor. Dent..

[4] Marachlioglou C.R.M.Z., Dos Santos J.F.F., Cunha V.P.P., Marchini L. (2010). Expectations and final evaluation of complete dentures by patients, dentist and dental technician. J. Oral Rehabil..

[5] Afrashtehfar K.I., Bryant S.R., Assery M.K.A., Isola G. (2020). Patient Satisfaction in Medicine and Dentistry. Int. J. Dent..

[6] Slade G.D., Spencer A.J. (1994). Development and evaluation of the Oral Health Impact Profile. Community Dent. Health.

[7] Larsson P., Bondemark L., Häggman-Henrikson B. (2021). The impact of oro-facial appearance on oral health-related quality of life: A systematic review. J. Oral. Rehabil..

[8] Larsson P., John M.T., Nilner K., Bondemark L., List T. (2010). Development of an Orofacial Esthetic Scale in prosthodontic patients. Int. J. Prosthodont..

[9] Larsson P. (2010). Methodological studies of orofacial aesthetics, orofacial function and oral health-related quality of life. Swed. Dent. J. Suppl..

[10] Wetselaar P., Koutris M., Visscher C.M., Larsson P., John M.T., Lobbezoo F. (2015). Psychometric properties of the Dutch version of the Orofacial Esthetic Scale (OES-NL) in dental patients with and without self-reported tooth wear. J. Oral. Rehabil..

[11] Campos L.A., Kämäräinen M., Silvola A.-S., Marôco J., Peltomäki T., Campos J.A.D.B. (2021). Orofacial Esthetic Scale and Psychosocial Impact of Dental Aesthetics Questionnaire: Development and psychometric properties of the Finnish version. Acta Odontol. Scand..

[12] Persic S., Milardovic S., Mehulic K., Celebic A. (2011). Psychometric properties of the Croatian version of the Orofacial Esthetic Scale and suggestions for modification. Int. J. Prosthodont..

[13] Shrout P.E., Fleiss J.L. (1979). Intraclass correlations: Uses in assessing rater reliability. Psychol. Bull..

[14] Enderlein G. (1988). Fleiss, J.L.: The Design and Analysis of Clinical Experiments. Wiley, New York—Chichester—Brislane—Toronto—Singapore 1986, 432 S., £38.35. Biom. J..

[15] Kline P. (2000). The Handbook of Psychological Testing.

[16] Larsson P., John M.T., Nilner K., List T. (2010). Reliability and validity of the Orofacial Esthetic Scale in prosthodontic patients. Int. J. Prosthodont..

[17] Pugača J., Urtāne I., Pirttiniemi P., Rogovska I. (2014). Validation of a Latvian and a Russian version of the Oral Health Impact Profile for use among adults. Stomatol. Balt. Dent. Maxillofac. J..

[18] Nunnally J.C., Bernstein I.H. (1994). Psychometric Theory.

[19] Sadrhaghighi A., Zarghami A., Sadrhaghighi S., Mohammadi A., Eskandarinezhad M. (2017). Esthetic preferences of laypersons of different cultures and races with regard to smile attractiveness. Indian. J. Dent. Res..

[20] Imafuku R., Nagatani Y., Shoji M. (2022). Communication Management Processes of Dentists Providing Healthcare for Migrants with Limited Japanese Proficiency. Int. J. Environ. Res. Public. Health.

[21] Khalid A., Quiñonez C. (2015). Straight, white teeth as a social prerogative. Sociol. Health Illn..

[22] Ahiaku S., Millar B.J. (2023). Maxillary Midline Diastemas in West African Smiles. Int. Dent. J..

[23] González E.L., Pérez B.P., Sánchez J.A.S., Acinas M.M.R. (2010). Dental aesthetics as an expression of culture and ritual. Br. Dent. J..

[24] Pinchi V., Barbieri P., Pradella F., Focardi M., Bartolini V., Norelli G.-A. (2015). Dental Ritual Mutilations and Forensic Odontologist Practice: A Review of the Literature. Acta Stomatol. Croat..

[25] Kostić M., Ignjatović A., Gligorijević N., Jovanović M., Đorđević N.S., Đerlek A., Igić M. (2023). Development and psychometric properties of the Serbian version of the Orofacial Esthetic Scale. J. Esthet. Restor. Dent..

[26] Lijmer J.G., Mol B.W., Heisterkamp S., Bonsel G.J., Prins M.H., van der Meulen J.H.P., Bossuyt P.M.M. (1999). Empirical Evidence of Design-Related Bias in Studies of Diagnostic Tests. JAMA.

[27] Rella E., De Angelis P., Nardella T., D’Addona A., Manicone P.F. (2023). Development and validation of the Italian version of the Orofacial Esthetic Scale (OES-I). Clin. Oral Investig..

[28] Paulina, Dhawan P., Jain N., Khan U. (2024). Validation, Adaptation and Assessment of Orofacial Esthetic Scale in Hindi Language. Indian J. Dent. Res..

[29] Karaokutan I., Senol H., Aksoy D., Ayvaz I., Cifci H. (2024). Development and psychometric properties of the Turkish version of the Orofacial Esthetic Scale. J. Esthet. Restor. Dent..

[30] Bimbashi V., Čelebić A., Staka G., Hoxha F., Peršić S., Petričević N. (2015). Psychometric properties of the Albanian version of the Orofacial Esthetic Scale: OES-ALB. BMC Oral Health.

[31] Reissmann D.R., Benecke A.W., Aarabi G., Sierwald I. (2015). Development and validation of the German version of the Orofacial Esthetic Scale. Clin. Oral Investig..

[32] Strajnic L., Bulatovic D., Stancic I., Zivkovic R. (2016). Self-perception and satisfaction with dental appearance and aesthetics with respect to patients’ age, gender, and level of education. Srp. Arh. Celok. Lek..

[33] Gavranović-Glamoč A., Kazazić L., Strujić-Porović S., Berhamović E., Džonlagić A., Zukić S., Jakupović S., Tosum Pošković S. (2021). Satisfaction and attitudes of the student population about dental aesthetics. J. Health Sci..

[34] Närhi L., Mattila M., Tolvanen M., Pirttiniemi P., Silvola A.-S. (2023). The associations of dental aesthetics, oral health-related quality of life and satisfaction with aesthetics in an adult population. Eur. J. Orthod..

